# Retraction: Comparison of Two Stump Closure Techniques in Laparoscopic Appendicectomy: A Single-Centre Prospective Cohort Study

**DOI:** 10.7759/cureus.r65

**Published:** 2023-01-13

**Authors:** Ashwin Vinod, Binoj ST, Nivedita S Nanda, Greeshma C Ravindran

**Affiliations:** 1 General Surgery, Amrita Institute of Medical Sciences and Hospital, Kochi, IND; 2 Gastrointestinal Surgery, Amrita Institute of Medical Sciences and Hospital, Kochi, IND; 3 Biostatistics, Amrita Institute of Medical Sciences and Hospital, Kochi, IND

This article has been retracted at the request of the authors due to several issues regarding inaccurate data and authorship.

Regarding concerns with data accuracy, the authors report that "There has been some mistake in data collection, as the data mentioned in the article and the total data of our department shows a slight mismatch. We are not sure at what level this mistake has happened and the only probable explanation could be duplication of data. The interns who have collected the data could have either collected the data of the same patient twice or could have made wrong entries into the master chart. Even though there is only a slight difference, this has significantly decreased the validity of the data."

Regarding mistakes in authorship, two authors were erroneously omitted from the author list. As stated by the authors, "The initial evaluation by our departmental board showed that Dr. George Mathews and Dr. Varun Venkatesh did not qualify for authorship. However, it has been sorted out among the team members and the department that Dr. Varun and Dr. George have contributed enough to warrant authorship. Hence omitting them from the authors list was another mistake made during article submission." Additionally, Dr. Binoj S T should have been designated as the corresponding author per the authors' institutional protocol.

The authors sincerely apologize for these unintentional mistakes and have requested that the article be retracted as a result. Upon a thorough review, the journal agrees with this course of action and therefore the article has been retracted.

